# Advancing Phishing Email Detection: A Comparative Study of Deep Learning Models

**DOI:** 10.3390/s24072077

**Published:** 2024-03-24

**Authors:** Najwa Altwaijry, Isra Al-Turaiki, Reem Alotaibi, Fatimah Alakeel

**Affiliations:** 1 Department of Computer Science, College of Computer and Information Sciences, King Saud University, Riyadh 11653, Saudi Arabia; ntwaijry@ksu.edu.sa; 2 Information Technology Department, College of Computer and Information Sciences, King Saud University, Riyadh 11451, Saudi Arabia; reem.awadh.d@gmail.com; 3 Department of Computer Science and Engineering, College of Applied Studies and Community Service, King Saud University, Riyadh 11495, Saudi Arabia; fyalakeel@ksu.edu.sa

**Keywords:** email phishing, deep learning, convolutional neural networks (CNN), LSTM, BiLSTM, BiGRU

## Abstract

Phishing is one of the most dangerous attacks targeting individuals, organizations, and nations. Although many traditional methods for email phishing detection exist, there is a need to improve accuracy and reduce false-positive rates. Our work investigates one-dimensional CNN-based models (1D-CNNPD) to detect phishing emails in order to address these challenges. Additionally, further improvement is achieved with the augmentation of the base 1D-CNNPD model with recurrent layers, namely, LSTM, Bi-LSTM, GRU, and Bi-GRU, and experimented with the four resulting models. Two benchmark datasets were used to evaluate the performance of our models: Phishing Corpus and Spam Assassin. Our results indicate that, in general, the augmentations improve the performance of the 1D-CNNPD base model. Specifically, the 1D-CNNPD with Bi-GRU yields the best results. Overall, the performance of our models is comparable to the state of the art of CNN-based phishing email detection. The Advanced 1D-CNNPD with Leaky ReLU and Bi-GRU achieved 100% precision, 99.68% accuracy, an F1 score of 99.66%, and a recall of 99.32%. We observe that increasing model depth typically leads to an initial performance improvement, succeeded by a decline. In conclusion, this study highlights the effectiveness of augmented 1D-CNNPD models in detecting phishing emails with improved accuracy. The reported performance measure values indicate the potential of these models in advancing the implementation of cybersecurity solutions to combat email phishing attacks.

## 1. Introduction

Phishing is a highly dangerous attack that targets individuals, organizations, and even nations. It involves social engineering tactics, where attackers impersonate legitimate entities to trick victims into disclosing sensitive information. According to the report released by the Anti-Phishing Working Group (APWG) [1], a staggering 1,286,208 phishing attacks were recorded in the second quarter of 2023. The report shows that 23.5% of all phishing attacks target the financial sector, making it the most attacked sector overall. Social engineering threats and attacks are the top concern for individuals and the second concern for many organizations [2]. Attackers use various deceptive techniques to gain access to sensitive information, such as login credentials, credit card details, or personal information. Social engineering is often the initial step in a cybercriminal’s attack plan, and in approximately 82% of cases, the spread of malware within a network begins with a phishing message [2]. Various communication methods are used by phishers to carry out their attacks, with the most common methods being email messages, social network messages, text messages, and phone calls.

Email phishing is a general term that refers to emails with malicious intent. A well-known example of an email phishing attack occurred in 2018 during the FIFA World Cup. Attackers targeted football fans by sending phishing emails promising recipients free tickets to Moscow, the hosting city of the 2018 FIFA World Cup. By tricking individuals into opening these phishing emails and clicking on embedded links, criminals successfully gained access to personal data from unsuspecting users.

Detecting phishing emails is essential to combating this type of attack and preventing cybercrime. Many organizations focus on strengthening their email security measures using a combination of methods. One way is the implementation of subdomain controls, which involves creating a separate domain specifically dedicated to email security to better protect against email-based attacks. In addition to this, user education and analysis of the history of phishing attacks are crucial for ensuring the security of individuals and organizations.

In the literature, classical approaches for phishing detection fall into two categories: *blacklists* and *signature-based* techniques [3]. *Blacklisting* is the act of making a list of suspicious resources used in previous phishing attacks. New suspicious contents can be checked against blacklists to confirm their validity. Unfortunately, due to the short lifespan of phishing links and the rapid creation of new ones, managing blacklists becomes difficult. Additionally, a single character change in the URL causes the website to be unrecognized by the blacklist. On the other hand, the *signature-based* approach focuses on utilizing features associated with the phishing act gathered from email addresses, links, URLs, and webpages in combination with rule setting to detect phishing attacks. Features of a newly accessed source are compared with with known phishing features identified from previous experiences. Although this approach is more efficient than list-based approaches and more effective in detecting zero-day attacks, it suffers from high false-positive rates.

Looking into the literature, we identify the following research gap. Traditional phishing detection approaches rely on human effort to analyze phishing email features, such as the sender, subject line, and contents. However, as the complexity of phishing attacks increases, these approaches are no longer adequate. Recently, approaches based on deep learning (DL) and machine learning (ML) have demonstrated the ability to overcome the limitations of traditional phishing detection methods [4]. ML algorithms can be used to train models that can detect phishing emails. Such models can learn phishing patterns and characteristics from large phishing datasets. Prior to training, important features related to phishing activity need to be first identified. This usually requires field expertise and a careful selection of essential features that result in efficient detection algorithms.

In contrast to ML algorithms, DL is capable of automatically extracting important features directly from raw data. Currently, deep neural networks are being used efficiently in several domains due to their state-of-the-art performance. In cybersecurity, researchers have demonstrated the potential of DL in tackling many cybersecurity problems [5]. However, more work is required to examine the robustness of deep neural networks for detecting email phishing [6]. DL algorithms, particularly convolutional neural networks (CNNs), long short-term memory (LSTM), and gated recurrent unit (GRU) models, showed promising results on different classification tasks including question classification, text categorization, and sentiment analysis and classification [7], among many others.

The problem context of the current study is phishing detection in emails. The problem is framed as a document binary classification task, treating every email as one document. Our goal is to classify emails into either *phishing* or *legitimate*. Our research question is how effective are deep learning models in detecting email phishing compared to existing methods? In particular, how effective is augmenting a CNN with recurrent layers in improving phishing detection performance? In order to achieve our goal, the objective of this study is to utilize DL architectures, including CNNs, LSTM, GRU, and and their variations. We define and measure the success of phishing email detection models in terms of improved performance in terms of standard metrics (precision, recall, accuracy, and area under the ROC curve). In this study, first, eight one-dimensional CNN models of various depths were trained using Spam Assassin [8] and Phishing Corpus [9] datasets. These models are collectively referred to as 1D-CNNPD models. Second, we augmented our base 1D-CNNPD model with LSTM and a GRU (and their variations) to train four additional models with the goal of improved performance. We call our augmented models Advanced 1D-CNNPD. LSTMs and GRUs are designed for sequential data and capturing temporal dependencies. Recent research suggests that augmenting a CNN with recurrent layers improves the phishing detection performance [10]. Deep neural networks are expected to enhance phishing email detection as they have superior abilities in terms of capturing hierarchical representations of features, considering both low-level and high-level abstractions. The performance of the twelve models for phishing detection was evaluated and compared with that of other similar models in the literature. In general, the performance of our models is comparable to state-of-the-art models. The 1D-CNNPD augmented with Bi-GRU outperformed advanced deep learning and machine learning phishing detection algorithms, achieving 100% precision, 99.68% accuracy, an F1 score of 99.66%, and a recall of 99.32%.

The main aim of this study was to investigate the potential of using deep learning for email phishing detection. In addition, we wished to examine the issue of deep learning model complexity in contrast with performance. Very deep models for natural language processing are complex and require vast resources and long training times to achieve excellent results, however, we hypothesize that such is not required for phishing detection. As such, we wish to study the effect of increasing model complexity, i.e., depth, for the problem of phishing detection. The contributions of this work are as follows: (1) we assessed the effects of varying the convolutional neural network depth on model performance in the context of phishing detection, (2) we developed a lightweight model that achieves excellent results, and (3) we recommend various interesting areas for future research.

**Figure 1:**
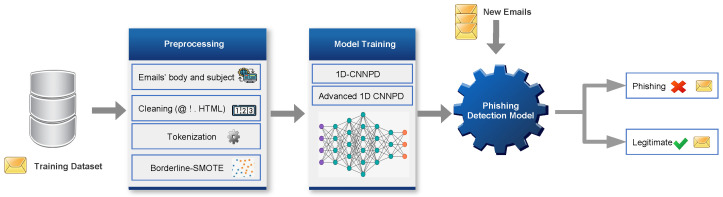
Email phishing classification system.

The proposed models can assist companies in providing a higher level of security against various types of email phishing attacks by detecting distinct features of such incidents and subsequently minimizing the occurrence of data breaches. Our models can be installed on the Edge Network Operation (ENO) of email. As shown in Figure 1, the framework receives a group of emails to evaluate and dispatches them to the email client.

The rest of the paper is organized as follows: In Section 2, we review the related literature on email phishing detection. Section 3 discusses the details of our 1D-CNNPD and Advanced 1D-CNNPD models. Then, we present experimental settings and results in Section 4. The impact and findings of this study are discussed in Section 5. Finally, Section 6 concludes the paper.

## 2. Related Work

Phishing detection continues to be a challenge for individuals and organizations. A high percentage of phishing attacks in recent years has driven the information security community to devise ways to detect and prevent the dangers of these attacks. According to the literature [11], anti-phishing solutions are grouped into software anti-phishing solutions and user awareness. Artificial intelligence approaches, such as machine learning, deep learning, and hybrid approaches, are widely integrated into software anti-phishing solutions [12]. In this section, we review the use of machine learning and deep learning to mitigate the risks of phishing attacks.

### 2.1. Machine Learning Approaches

Machine learning methods are widely used for the detection of phishing attacks [13]. Usually, the task is formulated into classification, clustering, or anomaly detection problems. In the case of classification, features relevant to the identification task are extracted from available datasets, and a machine learning model is trained.

In many studies, traditional machine learning algorithms, such as the C4.5 [14], Bayes Net [15], random forest [16], and SVM [17] algorithms, were used to accurately detect phishing attacks. One example of early work is the model presented by Ozarkar et al. [18] to classify spam emails. The authors used random forest and partial decision tree (PART) algorithms. Various feature selection techniques were used, such as chi-square and information gain. An accuracy of 96.181% was obtained in the study. Form et al. [19] used an SVM to categorize emails utilizing a collection of nine structure- and behavior-based features. The model had an accuracy of 97.25%. The main shortcoming of this work was the very small training set of 1000 emails. Han and Shen [20] adopted a semisupervised learning approach using a K-nearest neighbor attribute graph to detect spear phishing attacks. Four profiling features were used: origin, text, attachment, and recipient features. A dataset from Symantec’s enterprise email scanning service was used for training, with 1467 spear phishing emails and 4043 legitimate emails. Experimental results showed an F1 score of 90% and an FPR detection rate of 0.1 for known campaigns.

Other studies investigated the suitability of ensemble machine learning methods in the detection of phishing attacks. Hota et al. [21] proposed an ensemble machine learning based model with the remove replace feature selection technique (RRFST). Their aim was to limit the number of email phishing features to enhance machine learning performance. RRFST works by selecting a random feature from the set of feature space then replacing or keeping the feature based on the accuracy resulted from the ensemble of C4.5 and CART. After examining all features, the reduced feature set is fed into the ensemble, and the final accuracy is calculated. The result of its evaluation showed an increase in accuracy at 99.27% as compared to C4.5 and CART alone.

Yadav and Panda [22] trained three machine learning classification algorithms, decision trees (J48), random forest, and logistic regression, for the prediction of phishing emails. A total of 1000 emails from the Spam Assassin corpus were used to train the models, while 500 emails were used for testing and validation. Results showed that the best classification performance was obtained by the random forest algorithm, with a precision value of a 99%. With 15 feature sets, the reported accuracy of training and validation is 95.6% and 99.4%, respectively.

Machine learning has been combined with natural language processing (NLP) to effectively detect phishing emails. Contextual features [23], syntax features [15], and semantic features [24] have been applied in this field. Sahingoz et al. [4] investigated the potential of using several NLP-based word vectors and hybrid features with different machine learning algorithms in the detection of phishing webpages. NLP-based features used in this work are comparable to URL-based features used in other research works. Hybrid features used were a composition of NLP features and word vector features. The result of their investigation showed that random forest with NLP-based features achieved the highest accuracy, equal to 97.98%.

Recent work by Ghosh and Senthilrajan [25] classifies spam emails using machine learning classifiers and evaluates the performance of these classifiers. The authors implemented thirteen machine learning classifiers: the adaptive booster, artificial neural network, bootstrap aggregating, decision table, decision tree, J48, K-nearest neighbor, linear regression, logistic regression, naïve Bayes, random forest (RF), sequential minimal optimization, and SVM methods. According to the comparison of the accuracy of these models on the selected datasets, the RF outperformed other classifiers, as it achieved 99.91% for the Spam Corpus and 99.93% for the Spambase datasets. The naïve Bayes classifier achieved an accuracy of 87.63% for the Spam Corpus and 79.53% for the Spambase datasets.

Other recent work by Moutafis et al. [26] classified email messages as benevolent (“ham”) and malevolent (spam) by training ten machine learning models: the SVM, k-nearest neighbor, naïve Bayes, neural network, recurrent neural network, AdaBoost, random forest, gradient boosting, logistic regression, and decision tree methods. The testing was conducted on two datasets: Spam Assassin and Enron1. For the first dataset, the best performance was 99.51%, achieved by the NN, while the SVM achieved 99.38% for the second dataset.

While machine learning methods produce favorable results in phishing detection, they require carefully hand-crafted features. Most ML algorithms in the literature were applied to small datasets, which raised the question of scalability. In addition, they are vulnerable to zero-day phishing attacks that have unseen features for the classifier. An overview of machine learning algorithms used for phishing detection, along with their respective performance metrics, is shown in Table 1.

### 2.2. Deep Learning Approaches

Deep learning algorithms have demonstrated success in various fields, including computer vision [27], machine translation [28], and text categorization [29]. This success of deep learning algorithms encouraged cybersecurity researchers to use deep learning in many cybersecurity problems, including phishing detection. Deep learning techniques can automatically extract effective features from emails, eliminating the need for labor-intensive email feature extraction. Thus, they are able to capture a more thorough and comprehensive representation of information within the email text. Ra et al. [30] used word embedding and Neural Bag-of-Ngrams with deep learning methods to detect phishing emails. The authors experimented with various architectures, including CNN, RNN, LSTM, and MLP. The best accuracy value of 97.9% was obtained in the case of Neural Bag-of-Ngrams and MLP.

Fang et al. [10] proposed a phishing detection model based on recurrent convolutional neural networks (RCNNs). The proposed framework, THEMIS, combines an RCNN with multilevel embedding. The dataset used consisted of emails from the Enron Dataset, Spam Assassin, and First Security and Privacy Analytics Anti-Phishing Shared Task. The reported results showed that THEMIS outperforms LSTM and a CNN.

Alhogail and Alshabih [31] proposed a solution based on a graph convolutional network (GCN) and natural language processing of email body text. The introduced phishing email classifier was tested using the fraud dataset [32] which contains 3685 phishing emails and 4894 legitimate emails. Experimental results indicate that the model has an accuracy rate of 98.2% and a low false-positive rate of 0.015.

Doshi et al. [33] utilized features from email body and content to detect both spam and phishing emails. An ANN, RNN, and CNN were incorporated into a dual-layer architecture. The first layer classifies the phishing class, while the second classifies the spam class. Experiments were conducted on a total of 8218 emails from Phishing Corpus and Spam Assassin. Results show an accuracy, recall, precision, and F1 score of 99.51%, 99.68%, 99.5%, and 99.52%, respectively.

Dewis and Viana [34] used LSTM and MLP to to detect spam and phishing emails. Models were trained using Spam Assassin and and the Email Spam Classification dataset from Kaggle [35]. The proposed models obtained 99% average accuracy for the text-based datasets using LSTM and 94% for numerical-based datasets using MLP.

Alshingiti et al. [36] proposed a phishing detection system that combines CNN, LSTM, and LSTM-CNN networks. The authors collected a real-world dataset of phishing and legitimate emails and trained and evaluated their model on this dataset. The proposed system achieved a high accuracy and F1 score, demonstrating the effectiveness of the proposed approach.

Muralidharan and Nissim [37] introduced an approach for identifying malicious emails taking into account the entire email content, including the header, body, and attachments. An ensemble learning approach of various deep learning models was adopted. Various architectures were used, each for a specific email segment: a custom CNN and transfer learning model of BERT for the header, transfer learning of BERT for the body, and a custom CNN for attachments. Experimental evaluations were conducted on a set of emails from VirusTotal (20,037 benign and 12,639 malicious). Results show an AUC of 0.993, TPR of 0.9473, and FPR of 0.03.

Overall, the literature suggests the effectiveness of deep learning-based models, particularly the CNN, LSTM, LSTM-CNN, as well as the GRU [38], in phishing email detection. These models can potentially improve the accuracy and efficiency of phishing email detection systems, which can help prevent cyberattacks and protect sensitive information. An overview of deep learning algorithms used for phishing detection, along with their respective performance metrics, is shown in Table 2.

## 3. Materials and Methods

Our study focuses on using deep learning methods for the task of identifying phishing attacks. In particular, we are interested in the model design most suitable for email phishing detection. We also wish to answer the following question: are deeper (i.e., more complex models) needed for email phishing detection, or can simpler, more streamlined models perform detection suitably? In this section, we provide a detailed description of our dataset, the pre-processing steps, and the trained models that were chosen to perform our task.

### 3.1. Dataset Descriptions and Preprocessing

To create the proposed model, two publicly available datasets were used:

- The Phishing Corpus [9] is a widely used dataset for email phishing, comprising approximately 7315 phishing emails collected over a period of time.
- The Spam Assassin dataset [8] consists of legitimate and spam emails gathered by the Spam Assassin scheme. There are approximately 6047 email samples, in which there are 1897 spam emails and 4150 legitimate emails.

We selected these datasets as they are the most commonly used datasets in the literature [39]. More recent datasets, e.g., [40], offer no clear advantages over the more commonly used datasets, as they suffer from severe imbalance, as well as limited representation of more recent phishing attacks. Emails usually contain sensitive information, making collecting phishing email datasets difficult. Due to this limitation, publicly available datasets are also restricted, and only about 2278 phishing emails from the Phishing Corpus dataset were found in a public repository (Github). To train our models, we used a total of 6428 emails, consisting of 2278 phishing emails and 4150 legitimate emails. The email classification task classifies an email into one of two categories, either phishing or legitimate.

**Figure 2:**
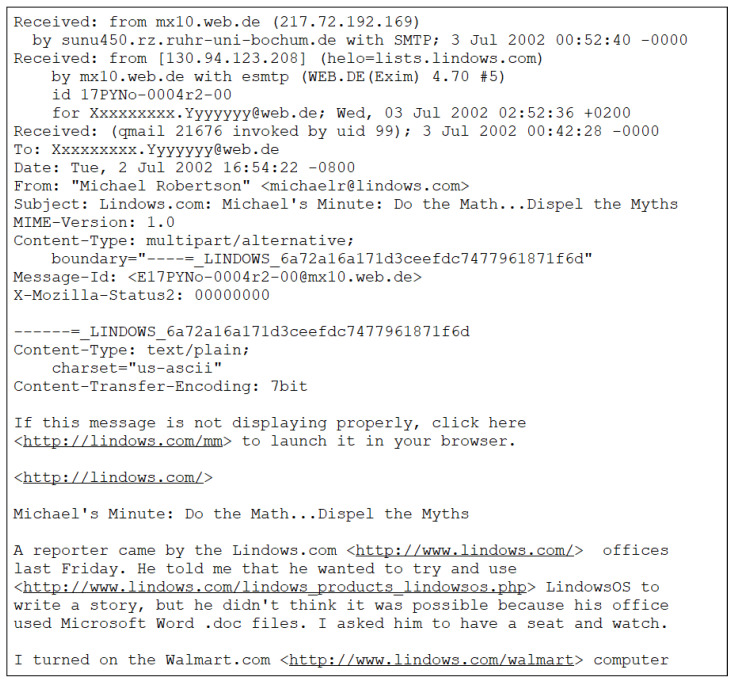
Sample email snippet.

The emails in the dataset are in email archive storage format, containing HTML tags, email header information, encoded images and files, text, and URLs. Figure 2 shows an example of an email message in the general format of the dataset. During preprocessing, the subject and body of the email were extracted, eliminating HTML tags, punctuation, encoded attachments, images, unnecessary spaces, IP addresses, email addresses, and stop words. Finally, URLs starting with http were replaced with [http].

The email text was then tokenized into individual words. A list of most common words in the dataset, defined as words that occur three times or more, was created. This list contains 22,105 words, from a total of 63,025 words in the dataset. This list was then used to further preprocess the emails, removing any words that are not in the list. Since our dataset was imbalanced, Borderline-SMOTE oversampling [41] was used to balance the dataset. The Borderline-SMOTE oversampling technique increases the smaller sample towards the class’s borderline and the closest neighbors in the same category. Research suggests that the efficiency of Borderline-SMOTE among many oversampling approaches has been verified [42].

### 3.2. Proposed Models

In this study, we used CNN-based models for email phishing attack detection. CNNs are among the best learning algorithms as they have superior abilities in terms of capturing hierarchical representations of features, considering both low-level and high-level abstractions. CNNs have demonstrated success in many cybersecurity problems, including phishing detection [36]. Compared to other deep learning models, a CNN requires fewer parameters, which reduces the complexity of the model and improves the learning process. In this study, we wish to create a simple lightweight model that can perform well in the phishing detection task. The model must have a relatively small number of parameters, short training times, and fast inference capabilities. We do this by building the simplest model possible, which we call 1D-CNNPD. This model is described in the next section. Next, we increase model size in a stepwise fashion, while calculating the performance of each, in order to find the best yet smallest model possible. Finally, we enhanced our best 1D-CNNPD model by incorporating LSTM and a GRU, along with their bidirectional variations. LSTMs and GRUs are specifically able to handle sequential data, allowing them to capture temporal dependencies effectively. Here, we provide a detailed description of our models.

#### 3.2.1. 1D-CNNPD Model

**Figure 3:**
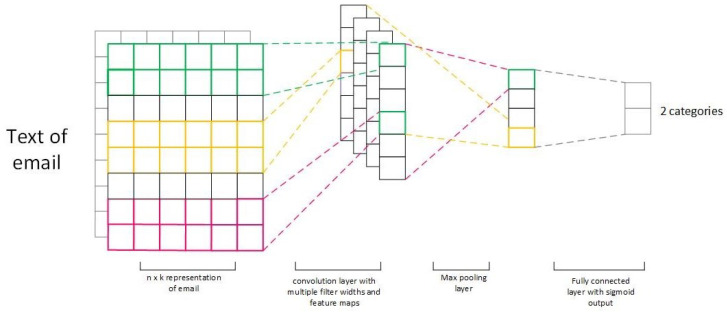
Document classification based on CNN.

CNNs are capable of extracting essential features in tokens or sequences of tokens regardless of the slight positional variations across diverse textual context. We view the phishing detection problem as a document classification task and adopted the CNN architecture for this task. Each email message was regarded as a single document. The CNN was trained to take a 1D input (email message) and output a document class (either phishing or legitimate). Our proposed architecture is illustrated in Figure 3. It consists of an embedding layer, convolutional layer, pooling layer, and a fully connected layer. The details of each layer are as follows:

**Input layer:** The input is a list of tokens representing each email. As emails have differing lengths, shorter emails are padded with zeros to a fixed maximum length, that of the longest email in the dataset.

**Embedding layer:** The embedding layer accepts the padded sequences of tokens and transforms them into real-valued vectors represented in a lower dimensional space. In this work, we used pretrained GloVe embeddings [43], as GloVe is able to capture both global and local statistics of a corpus. The embedding size used was 100.

**1D-CNN layer:** The word embedding vector matrix is then passed into a convolution layer, which extracts resilient features identifying a phishing email. These features are captured by convolutional filters. In our model, we studied various combinations of hyperparameters and architectures, see Table 3. As previously stated, we wish to study the effect of model size on model performance. A smaller model is faster and less expensive to train, as well as having a faster inference time. On the other hand, a model must be able to detect phishing accurately, otherwise its utility is reduced. Selecting hyperparameters for the model is another issue that must be dealt with. In order to find the best hyperparameters possible, we varied the filter size (1, 5, and 10), the number of filters used (100, 300, and 600), and the activation function tested (ReLU, Tanh, and Linear). For architectures, we varied the number of convolutional layers as shown in Table 3. We used batch normalization between layers to speed up the training process and avoid overfitting. The batch normalization layer was placed between the convolution layers and the pooling layer.

**Max-pooling layer:** In the next stage, max-pooling is applied to the output of the CNN layer. Max-pooling chooses the highest score among a matrix of features in the feature map. The pool size was set to 2. The major role of this layer is to reduce the volume of output vectors, and as a result, the output matrix is much smaller. In this study, we tested architectures with one or two pooling layers, as shown in Table 3.

**Fully connected layer (FC layer):** The output of the max-pooling layer is a matrix of consisting of 1D filters. The matrix is flattened then input into the following layer, which is a fully connected (FC) layer. The FC layer employs the sigmoid function, as shown in Equation (1):

(1) $$h_{\theta }\left(x\right)=\frac{1}{1+e^{-\theta^{T}x}}$$

**Output layer:** The output layer produces the email’s class, either phishing or legitimate, using the FC layer’s sigmoid activation function, since it has been shown to be effective among other test functions when used in the last layer for the text classification task [44].

**Hyperparameter tuning**: As mentioned in the previous sections, we studied various combinations of hyperparameters in order to select the optimal ones. The hyperparameters studied were selected based on the work of Zhang and Wallace [45]. The study suggested that the word embedding technique, kernel size, number of filters, and activation function used are the most important hyperparameters to consider while developing a CNN model for a document classification problem similar to that in this study. The kernel size was set to 1, 5, or 10, whereas the filter value was set to 100, 300, or 600, and finally, the activation functions ReLU, Tanh, and Linear were used. As the neural network training process is stochastic, outcomes might differ depending on the random weight initializers. To tackle the problem of randomness, we set the seed parameter value to a fixed value. Finally, we applied the early stopping regularization approach to stop the training process if the performance began to degrade. This also helps to minimize overfitting. Table 4 shows a summary of the hyperparameters.

#### 3.2.2. Advanced 1D-CNNPD

**Figure 4:**
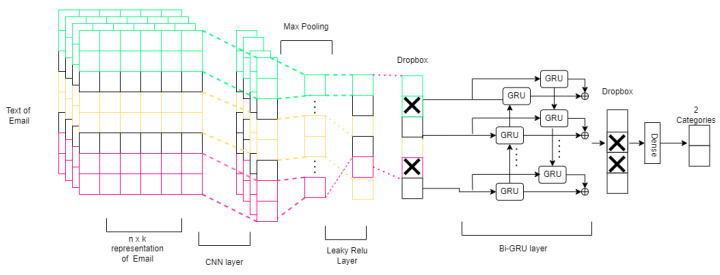
Advanced CNN with Bi-GRU and Leaky ReLU.

**Figure 5:**
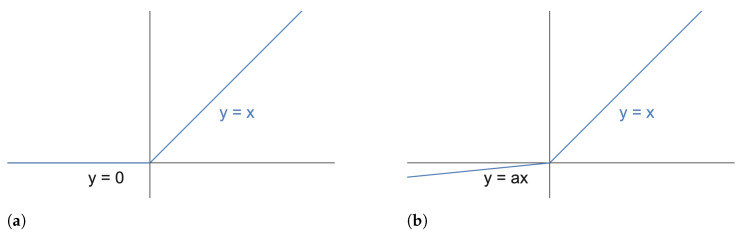
The main difference between Leaky ReLU and classical ReLU. (**a**) ReLU =max(0,x); (**b**) Leaky ReLU =max(αx,x).

To further enhance the performance of phishing detection, we augmented the best performing model of the 1D-CNNPD with LSTM and a GRU. In this section, we describe the modifications to the 1D-CNNPD model (the best performing model was base model 1 in Table 3). First, we discarded the fully connected layer and then added a ReLU layer with Dropout. Next, we experimented with four augmentations: adding an LSTM layer, a Bi-LSTM layer, a GRU layer, and a Bi-GRU layer. Figure 4 shows the Bi-GRU model. The details of our models are as follows:

**Leaky ReLU layer:** The Leaky ReLU layer with leak correction is a variation on the classic ReLU activation function. The function’s output has a small gradient toward the negative input. The main difference between the Leaky ReLU and classical ReLU is shown in Figure 5: the Leaky ReLU can be a negative value instead of a zero value, as shown in Equation (2).

(2) $$Leaky-ReLU\left(x\right)=\left\{\begin{array}{cc} \alpha x & x<0\\ x & x\geq 0 \end{array}\right.$$

where α is usually set as a small positive value, e.g., α=1e−2. The motivation for using this Leaky ReLU layer derives from its ability to handle negative input datasets.

**Long short-term memory (LSTM) layer:** LSTM is a type of RNN architecture that uses a number of gates and cell states to either remember or forget information over long sequences, enabling it to capture long-term dependencies in data. It helps to address the vanishing and exploding gradient problems that may occur in RNNs. Bidirectional long short-term memory (BiLSTM) is an extension of the LSTM architecture. It processes input sequences in both forward and backward directions, combining the output to create a comprehensive representation. This allows the network to capture information from past and future contexts simultaneously, enhancing its ability to model dependencies in sequential data.

**Gated recurrent unit (GRU) layer:** The GRU model preserves the original long short-time memory (LSTM) effect with a more straightforward structure, fewer parameters, and better accuracy. It has two gates, one for updating and one for resetting. The forward gate determines how much the previous hidden layer’s output affects the current layer: the more significant the value, the greater the influence. The reset gate controls how much of the previously hidden layer information is ignored. The bidirectional gated recurrent unit (Bi-GRU) is divided into two unidirectional GRUs: the first is an output of the hidden layer state forward →, as shown in Equation (3), and the second ← is the backward hidden layer state shown in Equation (4).

(3) $$h_{t}^{\to }={\mathrm{GRU}}_{\mathrm{FWD}}\left(x_{t},h_{t-1}\right)$$

(4) $$h_{t}^{\leftarrow }={\mathrm{GRU}}_{\mathrm{BWD}}\left(x_{t},h_{t+1}\right)$$

where $h_{t}^{\to }$ is the forward GRU state, and $h_{t}^{\leftarrow }$ is the backward GRU state and signifies the operation of concatenating two vectors. We included the Bi-GRU rather than the Bi-LSTM for two reasons: First, the Bi-GRU has two gates, which is appropriate for the size of our small dataset. Second, we fit fewer parameters for our small dataset and save time.

It is important to note that the incorporation of machine learning models into real email security systems goes beyond software application embedding. In the real world, the process needs to be continuously monitored and fine-tuned to ensure that the model can manage the volume of user requests while producing results that are impartial and unambiguous. The deployment of a model involves numerous teams, tools, and components, making it a complicated task. Data scientists, MLOps engineers, and developers work together on this task. The deployment strategy must include real-time unstructured data extraction and processing; storage requirements identification; API, tool, and software environment setup; prediction timeframe estimation; hardware or cloud requirements to meet computational demands; and pipeline setup for ongoing training and parameter tuning.

## 4. Results

### 4.1. Experimental Settings

The proposed models were implemented using Tensorflow, an open source machine learning library, utilizing Keras. The Talos hyperparameter tuning library [46] was used to select the best performing model. Experiments were carried out using GPUs running on the Google Colab environment. To evaluate our models, we split the dataset into 70% for training and 30% for testing. The training set was further split into 70% for training and 30% for validation.

### 4.2. Performance Measures

To evaluate our phishing email classification models, we calculated accuracy, recall, precision, and F1 score. Furthermore, since our dataset was originally imbalanced, we calculated the ROC AUC to test the effectiveness of the oversampling method used. The definition and calculation used for each measure is detailed as follows:

- Accuracy measure: the percentage of correctly classified emails.

(5) $$\mathrm{Accuracy}=\frac{TP+TN}{TP+TN+FP+FN}$$

- Recall: the fraction of phishing emails correctly classified as phishing out of the total number of phishing emails.

(6) $$\mathrm{Recall}=\frac{TP}{TP+FN}$$

- Precision: the fraction of phishing emails correctly classified as phishing out of all the samples predicted as phishing emails.

(7) $$\mathrm{Precision}=\frac{TP}{TP+FP}$$

- F1 score: the harmonic mean of precision and recall.

(8) $$F-\mathrm{Score}=\frac{2\times \mathrm{Precision}\times \mathrm{Recall}}{\mathrm{Precision}+\mathrm{Recall}}$$

where TP (true positives) is the total number of emails that are correctly classified as phishing emails, FP (false positives) represents the total number of emails that have been incorrectly classified as phishing emails, FN (false negatives) represents the total number of emails that have been incorrectly classified as non-phishing, and finally TN (true negatives) represents the total number of emails that have been correctly classified as non-phishing.

Finally, the *receiver operating characteristic area under the curve* (ROC AUC) score is the probability of scores achieved by the classifier to rank a randomly chosen phishing email higher than a randomly chosen legitimate email. This measure can be calculated by computing the area under the ROC curve.

### 4.3. Experimental Results

The purpose of our experiments was to evaluate the ability of CNN-based models to detect phishing emails. We experimented with standalone CNN models, the 1D-CNNPD, and a CNN model augmented with LSTM and a GRU, the Advanced 1D-CNNPD. We utilized two benchmark datasets, Phishing Corpus [9] and Spam Assassin [8], to train our models. The performance results were compared with results of traditional machine learning approaches in the literature and also with similar deep learning models.

#### 4.3.1. 1D-CNNPD Models

To find the best performing model among the different network architectures under study, the Talos scan function was used to perform 216 permutations over different architectures of CNNs. The models were trained on the training set and evaluated on the validation set to select the best hyperparameters for the different CNN architectures. Table 5 shows the results of the best hyperparameters for the different network architectures that were under study. The reported hyperparameters were chosen based on the best validation accuracy achieved during the tuning process.

Next, in Table 6, we report the mean values of the evaluation measures for the test set based on 30 rounds of running each model in order to minimize bias in results. Among the eight trained models, the best results were achieved by the first model in terms of accuracy, precision, recall, and F1. We observe that model 4, which includes two convolutional layers, has comparable performance to model 1. Both of these models also have a small standard deviation for the F1 score, indicating stable performance. Arch. 1 has one convolutional layer that requires 100×300+300=30,300 trainable parameters, while Arch. 4 has two convolutional layers requiring 100×600+600=60,600 parameters for the first layer, and 600×600+600=360,600 parameters for the second layer, for a total of 421,200 parameters. The minuscule amount of trainable parameters for our selected model, Arch. 1, shows that it is indeed a lightweight model.

Our results highlight the widely debated topic of whether deeper models can produce more precise outcomes. In our experiments, we observe that increasing the number of convolutional layers in the network (greater than 2) produces a negative effect on the model performance as it encourages overfitting, as seen from the ROC AUC measurement.

Next, to further verify the effectiveness of our model, we compared our best performing model (base model 1) with traditional ML models tackling the same problem. Specifically, we compared our model with models that have also used the Spam Assassin and Phishing Corpus email phishing datasets. Table 7 presents the results of our best model (model 1) compared with previous traditional ML models results in the literature. We can see that our model shows comparable results to other ML approaches. This shows that a CNN with convolutional filters is capable of extracting resilient phishing features without the need for a manual feature extraction process. It is also observed from the table that machine learning algorithms can exhibit high performance when trained using a wide range of handcrafted features. According to the literature, the most effective handcrafted features for email phishing detection can be grouped into external features, body-based features, URL-based features, header-based features, and sender-based features [47]. Despite the high performance, handcrafting features is a laborious process that can be very time-consuming and error-prone.

Although in this study we focus on an email’s body and subject, high performance is observed, indicating that there is room for improvement with the inclusion of more email information. In addition, handcrafting features is not required.

#### 4.3.2. Advanced 1D-CNNPD

Our next experiment was designed to study whether the performance of our best performing 1D-CNNPD model (base model 1) can be improved when augmented with LSTM and a GRU, and their bidirectional counterparts. In Table 8, we show the performance measures of our proposed augmentations, the 1D-CNNPD base model, and other similar models in the literature. First, we observe that, in general, the augmentations improve the performance of the 1D-CNNPD base model. In particular, the 1D-CNNPD with Bi-GRU yields the best results. It is also observed that augmentations with bidirectional LSTM and a GRU have improved performance over their unidirectional variations as they are able to capture long-range dependencies.

Second, we compared our results to DeepAnti-PhishNet [30], which also uses LSTM and THEMIS [10] and is based on an RCNN. Our choice of deep learning models for comparison is based on studies that share a similar problem formulation to ours. Our dataset is smaller than the DeepAnti-PhishNet dataset, and the LSTM layer typically performs better with larger datasets. It is important to note that DeepAnti-PhishNet is biased towards the classification of legitimate emails, since it was trained using an imbalanced corpus. Results indicate that the 1D-CNNPD with Bi-GRU augmentations performs better than both DeepAnti-PhishNet and THEMIS. We additionally contrast our most effective model with the dual-layer architecture introduced by [33] and the CNN model with Continuous Bag of Words (CBOW) word embedding [6]. The dual-layer design utilizes both email content and body. Each layer incorporates a pre-trained model responsible for classifying data instances into their respective classes. The CNN model with CBOW is a simple model with two convolutional layers and two max-pooling layers. The results in Table 8 show the improved performance of our lightweight model, 1D-CNNPD with Bi-GRU, over the other two models.

## 5. Discussion

The main goal of this study was to investigate how email phishing detection could be improved using DL-based approaches. Email phishing detection using deep learning is rapidly evolving. This stems from the potential of deep learning to overcome the limitations of traditional methods and enhance the accuracy of detection. CNN, LSTM, and GRU architectures are capable of analyzing the contents and structure of phishing emails, as demonstrated in various studies [3]. However, a major success measure for any phishing detection model is the ability to detect zero-day phishing attacks with low false-positive rates.

A total of twelve CNN-based models were trained to detect phishing emails. Our problem is formulated as a binary classification problem of documents, where it is required to classify each email (document) as being a phishing or legitimate email. Our results demonstrate that 1D-CNNPD models are very robust in extracting features of resilient phishing without using any hand-engineering feature extraction method. Experimenting with various depths brought to our attention the widely discussed issue of whether deeper models can yield more accurate results. Our findings show that performance degrades as the number of convolutional layers increases. This could be the result of model overfitting. We note that in our experiments, some deeper models exhibit excellent performance; nevertheless, they display a higher standard deviation score, suggesting that their efficacy may not be consistent across various iterations. Our observation aligns with findings in the literature, indicating that increasing model depth tends to exhibit an initial improvement in performance followed by degradation [53,54].

Historically, traditional machine learning algorithms have been extensively employed to address phishing detection and continue to be in use today. Despite being categorized as shallow learners, they proved to be effective in certain settings. This research illustrates the superiority of deep learning over traditional shallow learning in the realm of phishing detection. The intricate architectures of deep learning models allow automatic extraction of features and contribute to a more effective learning process compared to their shallow counterparts. Two well-known datasets were used to train our models: Spam Assassin and Phishing Corpus datasets. Despite being collected between 2004 and 2007, they remain the most widely used in the literature [39]. Recently, there have been efforts to collect phishing datasets that mimic the evolution of phishing attacks. The dataset compiled by Bountakas and Xenakis [40] contains 35,511 emails. However, only 3460 are phishing emails, of which 1472 were collected between 2015 and 2020. Although it is a more recently published dataset, it is severely imbalanced, and the phishing emails were not recent. As such, it provides no clear advantages for us over the more commonly used datasets. The lack of representative datasets in the field of phishing emails is still an ongoing concern for researchers.

Our results indicate that the 1D-CNNPD with Bi-GRU augmentations outperforms DeepAnti-PhishNet in terms of accuracy. It is crucial to highlight that DeepAnti-PhishNet exhibits a bias toward classifying legitimate emails due to training on an imbalanced corpus. In comparison to THEMIS, the 1D-CNNPD with Bi-GRU combination demonstrates a higher F1 score. For highly concealed phishing emails, where the email body exhibits an extremely high similarity to legitimate emails, the attention mechanism in THEMIS assigns a higher weight to the email header than to the email body. During THEMIS training, email header information from an open-source dataset was utilized, which could be the reason for the improved accuracy.

In practice, the implementation of email phishing detection can be tailored to the specific requirements and constraints of the system. Two common deployment scenarios are at the email server or at the edge of the network. Implementing the detection component directly on the email server enables real-time analysis and the immediate identification of potential phishing emails. Alternatively, deploying it at the network’s edge provides an added layer of security, with all incoming emails passing through the edge device for analysis before being forwarded to the email server or the recipient’s mailbox.

The phishing detection technologies that are now in use have a number of practical limitations. Zero-day attacks present a serious problem as they can continuously introduce new phishing patterns that the system has never seen before. Another challenge is spear phishing, which involves highly targeted and customized attacks that do not match known phishing patterns. Social engineering techniques are becoming more common, which further complicates things by taking advantage of human vulnerabilities and making it harder for automated systems to detect malicious intent based only on technical factors. Although AI-based systems have demonstrated tremendous success in many fields, an important challenge that remains to be addressed is algorithmic bias resulting from limited datasets. In the context of phishing detection, training datasets are inherently limited, exhibiting an imbalanced class distribution. Malicious and phishing emails are typically underrepresented, and this imbalance may lead to biased predictions with potentially unwanted consequences.

Despite all developments in deep learning, research shows that it has not been extensively studied in the context of phishing detection. One area to investigate is the effect of hyperparameter fine-tuning algorithms to ensure the robustness of deep learning architectures for phishing detection. Many optimization algorithms, such as random search, grid search, and Bayesian optimization, could be explored. Another requirement for future phishing detection models is to have representative datasets, as class-imbalanced datasets can lead to poor and biased detection performance. Although many class-imbalanced algorithms exist, obtaining reliable and precise results is still challenging. This is particularly challenging in the case of multi-label and multi-class phishing detection. Thus, future research needs to focus on adapting class-imbalanced algorithms to the ever-evolving nature of phishing patterns. Emerging technologies pose more challenges in the detection of cyberthreats, including phishing attacks. Cybercriminals are utilizing AI technologies, such as generative AI, to create content that resembles a known contact’s tone and style, making it more difficult for phishing detection solutions to recognize. Additionally, phishing attacks can become more targeted as a result of the growing amount of behavioral data being collected. Attackers can track user activity and execute customized attacks through AI automation. Finally, deep learning is sometimes thought of as a “black box”, which means that it might be difficult to analyze or comprehend the inner workings of the model and the particular features that influence its predictions. However, AI explainability is an emerging research topic and there is ongoing work to explore the explainability of deep learning methods in the context of phishing detection, a topic that is worth investigation.

Phishing attacks present implications on various levels, including theoretical, managerial, and social. Many theoretical frameworks have been proposed to predict phishing susceptibility. Research suggests that individual characteristics like age, gender, and technological proficiency do not correlate with a person’s susceptibility to phishing. Rather, training and anti-phishing education can help control a user’s response to a phishing attack. Institutional and business IT managers should be mindful of the harmful aspects of phishing attacks to organizations that would result in sensitive data breaches, loss of data and intellectual property, reputation damage, customer churn, and monetary losses. Protecting organizations from phishing attacks requires a combination of countermeasures, including employee education, technical solutions, and compliance with policies and relevant laws. Phishing attacks have far-reaching societal effects since they diminish society’s trust in using technology, making people less confident in conducting critical transactions online or assisting others.

## 6. Conclusions

Although phishing attacks were introduced two decades ago, they are still used to gain personal information, user credentials, and credit card details. In this work, we addressed email phishing because emails are the most common entry point for phishers to initiate attacks. We present a total of twelve DL models based on a CNN for phishing detection. Our results indicate that the combination of a CNN with Bi-GRU is able to accurately detect phishing emails. Bi-GRU is used to save time without sacrificing performance. The result of the Advanced 1D-CNNPD with Leaky ReLU and Bi-GRU achieved 100 % precision, 99.68% accuracy, an F1 score of 99.66%, and a recall of 99.32%. A huge number of solutions have been proposed to encounter phishing attacks. However, phishers always succeed in discovering new vulnerabilities in the developed solution. This battle between software developers and phishers has no end. Our work can be extended in many ways. We suggest using *Generative Adversarial Networks* as a solution by applying Bi-GRU as the generator layers and 1D-CNN as the discriminator layers. In addition, more information could be used in model training, such as email headers, to further improve performance. Due to the lack of real phishing datasets, it is important to investigate dataset expansion approaches, such as text augmentation approaches.

## Figures and Tables

**Table 1:** Summary of machine learning algorithms for phishing detection.

Ref.	Algorithm	Dataset	Evaluation
[14]	C4.5	WestPac emails	Acc: 99%
[15]	Bayes Net	Phishing Corpus and SpamAssassin	Acc: 92%
[16]	RF	Phishing Corpus and SpamAssassin	Acc: 97%, FP 0.60%
[17]	SVM	Phishery and 2007 TREC Corpus	Acc: 98.2%
[18]	RF	Enron	Acc: 96.18%
[18]	PART	Enron	Acc: 95.09%
[19]	SVM	Phishing Corpus and SpamAssassin	Acc: 97.25%
[26]	NN	Spam Assassin	Acc: 99.51%
[26]	SVM	Enron1	Acc: 99.38%
[20]	KNN	Symantec’s enterprise emails	F1: 90%, FPR: 0.1
[21]	Ensemble (C4.5 and CART)	Khonji’s anti-phishing website	Acc: 99.27%
[4]	RF and NLP	Phishing webpages	Acc: 97.98%
[25]	RF	Spam Corpus	Acc: 99.91%

**Table 2:** Summary of deep learning algorithms for phishing detection.

Ref.	Algorithm	Dataset	Evaluation
[30]	N-grams and MLP	IWSPA-AP 2018 dataset	Acc: 97.9%
[10]	RCNN	Enron, SpamAssassin	Acc: 99.84% F1: 99.33%
[31]	GCN	Fraud dataset	Acc: 98.2%, FPR: 0.015
[33]	ANN, RNN, CNN	Phishing Corpus and Spam Assassin	Acc: 99.51%, F1: 99.52%
[34]	LSTM	Spam Assassin and Kaggle	Acc: 99%
[36]	LSTM-CNN	URL 2016 Dataset	Acc: 97.6, F1: 97.6
[37]	CNN and BERT	VirusTotal	AUC: 0.993, TPR: 0.9473

**Table 3:** The different CNN architectures used in our experiments.

Arch.	Network Structure
1	emb–conv–maxPool–FC
2	emb–conv–conv–maxPool–FC
3	emb–conv–conv–conv–maxPool–FC
4	emb–conv–maxPool–conv–maxPool–FC
5	emb–conv–conv–maxPool–conv–maxPool–FC
6	emb–conv–conv–conv–maxPool–conv–maxPool–FC
7	emb–conv–conv–conv–maxPool–conv–conv–maxPool–FC
8	emb–conv–conv–conv–maxPool–conv–conv–conv–maxPool–FC

**Table 4:** Summary of hyperparameters.

Hyperparameter	Value
Seed	42
Loss	Binary_crossentropy
Number of Layers	1, 2, 3, 4, 5, or 6
Kernel Size	1, 5, or 10
Filters	100, 300, or 600
Activation Function	ReLU, Tanh, or Linear
FC layer Activation Function	Sigmoid
Optimizer	Adam optimizer, learning rate of 0.001
Early Stopping	monitor = ‘val_loss’, patience = 2
Batch Size	32
Epochs	100

**Table 5:** Results of hyperparameter optimization.

1D-CNNPD	Activation	Kernel Size	Filters
Arch. 1	*ReLU*	1	300
Arch. 2	*Tanh*	1	600
Arch. 3	*Linear*	1	600
Arch. 4	*ReLU*	5	100
Arch. 5	*Linear*	1	100
Arch. 6	*Linear*	1	300
Arch. 7	*Linear*	5	100
Arch. 8	*Linear*	5	300

**Table 6:** Experimental results for each architecture of our base model, 1D-CNNPD.

1D-CNNPD	Accuracy	Precision	Recall	F1	STD F1	ROC AUC
Arch. 1	**98.87**%	**97.19%**	**99.59%**	**98.38%**	**0.09%**	**99.85%**
Arch. 2	79.67%	77.64%	89.30%	77.91%	18.28%	82.02%
Arch. 3	70.86%	68.96%	82.25%	67.42%	22.41%	73.64%
Arch. 4	98.73%	97.16%	99.20%	98.17%	0.13%	99.76%
Arch. 5	90.01%	88.48%	92.08%	87.76%	13.95%	92.11%
Arch. 6	65.04%	67.96%	81.42%	62.34%	24%	69.16%
Arch. 7	96.14%	95.19%	94.41%	93.90%	10.73%	96.50%
Arch. 8	34.42%	34.35%	100%	51.14%	0	50.08%

**Table 7:** The performance of the proposed model compared with related machine learning work, where ML is machine learning, P is phishing, L is legitimate and S is spam.

ML Algorithm	Dataset (P, L, S)	Feature Count	Results
C4.5 [14]	46,525/613,048	7	Acc. 99%
J48 and SVM [17]	5000/5000	30	Acc. 99.7%
C5.0 [48]	4563/4202/1895	22	Acc. 97%
Bayes Net [15]	4594 total	7	Acc. 92%
Random forest [16]	4116/4150	47	Acc. 97% FP 0.60%
PLSA [49]	400,000 total	200 topics	Acc. 97.7%
ECM and ECMc [50]	4300/6000	21	Acc. 98%
SVM, AdaBoost, [51]	N/A	21	Acc. 97% FP 2%
and naïve Bayes [51]			FN 9%
Neural networks and	4559/4559	50	Acc. 98.6%
reinforcement learning [52]			F1 98.64%
1D-CNNPD	2279/4150	Auto	Acc. 98.87% F1 98.38%

**Table 8:** The progress of the proposed model compared with deep learning approaches.

Deep Learning Algorithms	Dataset (Phishing/Legitimate/Spam)	Feature Counts	Results	
			**Acc.**	**F1**
DeepAnti-PhishNet [30]	612/5088 train 4300 test	Body	99.1%	-
THEMIS [10]	4999/7781	Body and header	99.84%	99.33%
Dual-layer CNN [33]	2664/5554	Body and content	99.51%	99.52%
CNN [6]	14,950/3416	Body	96.34%	-
1D-CNNPD	2279/4150	Body and subject	98.87%	98.38%
1D-CNNPD with LSTM	2279/4150	Body and subject	99.23%	99.20%
1D-CNNPD with Bi-LSTM	2279/4150	Body and subject	99.34%	99.31%
1D-CNNPD with GRU	2279/4150	Body and subject	99.01%	99.66%
1D-CNNPD with Bi-GRU	2279/4150	Body and subject	99.68%	99.66%

## Data Availability

The Spam Assassin Dataset can be found at https://spamassassin.apache.org/old/publiccorpus/20050311_spam_2.tar.bz2, accessed on 5 May 2023.The Phishing Corpus can be found at https://academictorrents.com/details/a77cda9a9d89a60dbdfbe581adf6e2df9197995a, accessed on 5 May 2023.

## Abbreviations

The following abbreviations are used in this manuscript:

1D-CNNPD	One-Dimensional Convolutional Neural Network for Phishing Detection
AUC	Area Under the Curve
CNN	Convolutional Neural Network
GRU	Gated Recurrent Unit
LSTM	Long Short-Term Memory
ROC	Receiver Operating Characteristic
SMOTE	Synthetic Minority Oversampling Technique
SVM	Support Vector Machine
